# Early childhood psychological factors and risk for bedwetting at school age in a UK cohort

**DOI:** 10.1007/s00787-015-0756-7

**Published:** 2015-08-21

**Authors:** Carol Joinson, Sarah Sullivan, Alexander von Gontard, Jon Heron

**Affiliations:** School of Social and Community Medicine, University of Bristol, Oakfield House, Oakfield Grove, Clifton, Bristol, BS8 2BN UK; Epidemiology and Health Services Research, CLAHRC West, Lewins Mead, Bristol, UK; Department of Child and Adolescent Psychiatry, Saarland University Hospital, 66421 Homburg, Germany

**Keywords:** Bedwetting, Psychological factors, Cohort study, ALSPAC

## Abstract

There is evidence for a link between psychological factors and bedwetting, but the direction of this association is unclear. Using data on 8769 children from the Avon Longitudinal Study of Parents and Children, we examined whether difficult temperament (Toddler Temperament Scale at 24 months; Emotionality Activity Sociability Questionnaire at 38 months) and psychological problems (Revised Rutter Parent Scale for Preschool Children at 42 months) are linked to bedwetting at school age. We examined the association between these risk factors and different patterns of bedwetting from 4 to 9 years using multinomial regression. Difficult temperament and psychological problems in early childhood were associated with increased odds of bedwetting at 4–9 years. The strongest associations were most often found for the pattern of bedwetting that was both frequent (at least twice a week) and persistent (up to age 9) e.g. the temperament traits of ‘adaptability’ and ‘mood’ were associated with a 33 % increase (95 % confidence interval = 1.14–1.55) and a 27 % increase (1.10–1.47) respectively in the odds of persistent and frequent bedwetting per one standard deviation increase in risk score. Early behaviour problems (e.g. conduct problems [1.43 (1.25, 1.63)] and hyperactivity [1.29 (1.11, 1.50), *p* < 0.001]) were also associated with frequent and persistent bedwetting, but there was less evidence that early emotional difficulties were risk factors for bedwetting. Adjustment for confounders did not alter these conclusions. The presence of difficult temperament and behaviour problems in early childhood might help to identify children who will continue to experience bedwetting at school age.

**Electronic supplementary material:**

The online version of this article (doi:10.1007/s00787-015-0756-7) contains supplementary material, which is available to authorized users.

## Introduction

The prevalence of bedwetting decreases with age [1], with most children attaining nighttime bladder control by around 4–6 years [2]; however, a significant number of children are still wetting the bed at school age. For example, bedwetting was reported in 15.5 % of 7-year olds in the Avon Longitudinal Study of Parents and Children (ALSPAC) [3]. Nocturnal enuresis is the term used by the International Children’s Continence Society to describe bedwetting in children aged 5 years or older after ruling out organic causes [4]. The aetiology of bedwetting is believed to be multifactorial, involving a complex interrelationship of biological, developmental, genetic, psychosocial and environmental factors. The relative importance of each type of risk domain is unknown and the extent to which each one contributes to the risk for bedwetting is likely to differ between children.

There is evidence for a link between bedwetting and psychological factors, but the direction of this association is unclear since the majority of earlier studies are cross-sectional—see [5] for a review. Cross-sectional studies are potentially biased since parents of children who wet the bed may be negatively predisposed to their children and, hence, more likely to report psychological problems. Distress and intolerance are not uncommon among parents of children with nocturnal enuresis [6] and this could result in increased levels of emotional and behavioural symptoms in children.

Potential explanations have been proposed to explain the observed association between bedwetting and psychological factors [7]. The association could have a common underlying neurobiological basis such as developmental delay, which is linked to both bedwetting [8, 9] and psychological problems [10]. For instance, the high co-occurrence of enuresis and ADHD is based on complex neural networks including cortical, subcortical and brainstem regions [11].

Psychological problems could be a consequence of bedwetting due to the distress and loss of self-esteem often reported by sufferers [12]. The assertion that psychological problems are induced by bedwetting is supported by studies reporting an improvement in these problems following successful treatment for bedwetting [13, 14].

A further explanation is that psychological problems could be a risk factor for bedwetting. Earlier studies have shown that behaviour problems [15] and stressful life events [16] sometimes precede relapses in bedwetting. There is evidence that early difficult temperament is a precursor to behaviour and emotional problems in children [17] and bedwetting is linked to elevated rates of these problems. However, to our knowledge there are no studies examining whether difficult temperament and psychological problems in early childhood are linked to subsequent problems attaining nighttime bladder control.

In order to properly examine this, a prospective cohort study is needed with data on temperament and psychological problems in early childhood (i.e. before the age at which most children attain nighttime bladder control) and subsequent data on bedwetting at school age. This would address potential biases inherent in earlier studies since parents are reporting difficult temperament and psychological problems before bedwetting is normally considered unusual or problematic.

We use data from a large UK cohort (ALSPAC) to examine whether difficult temperament and psychological problems in early childhood are associated with bedwetting at school age. Previously, we used longitudinal data derived from parental reports of frequency of bedwetting at ages 4–9 years in almost 11,000 children from the ALSPAC cohort to identify different patterns (latent classes) of typical and atypical development of nighttime bladder control [18]. We now build on this work by using these latent classes to examine whether difficult temperament and psychological problems (assessed at 2–3 years) precede bedwetting assessed at 4–9 years.

## Methods

### Participants

The sample comprised participants from the Avon Longitudinal Study of Parents and Children. Detailed information about ALSPAC is available on the study website (http://www.bristol.ac.uk/alspac), which includes a fully searchable dictionary of available data (http://www.bris.ac.uk/alspac/researchers/data-access/data-dictionary). Pregnant women resident in the former Avon Health Authority in south-west England, having an estimated date of delivery between 1/4/91 and 31/12/92 were invited to take part, resulting in a cohort of 14,541 pregnancies and 13,973 singletons/twins (7217 boys and 6756 girls) alive at 12 months [19]. Ethical approval for the study was obtained from the ALSPAC Law and Ethics committee and local research ethics committees.

### Latent classes of bedwetting

At ages 4^1^/_2_, 5^1^/_2_, 6^1^/_2_, 7^1^/_2_ and 9^1^/_2_ years (hereafter referred to as 4–9 years) parents were asked “How often usually does your child wet the bed?” and were given the options ‘never’; ‘less than once a week’; ‘about once a week’; ‘2–5 times a week’; ‘nearly every night’; ‘more than once a night’. Previously, we used these repeated measures data to identify trajectories of typical and atypical development of nighttime bladder control using longitudinal latent class analysis (LLCA) [18]. LLCA is a useful tool to examine developmental trajectories of disorders because it provides an empirical means of summarising large amounts of data and identifying clusters of individuals (latent classes) with typical and atypical courses of development. We derived an ordinal measure by collapsing the bedwetting frequency data into a three-level variable indicating no current bedwetting, infrequent bedwetting (less than once a week or about once a week) and frequent bedwetting (2–5 times a week, nearly every night or more than once a night). The latter category corresponds to the frequency of bedwetting required for a DSM-V diagnosis of nocturnal enuresis. LLCA examines response strings that describe each child’s longitudinal pattern of frequency of bedwetting (no bedwetting, 0; infrequent bedwetting, 1; frequent bedwetting, 2) from 4 to 9 years. For instance, the data for a child who attains nighttime bladder control at an early age may be “10,000”; a child who experiences delayed attainment (at around 6 years) might have the response “21,000” and the response pattern for a child with persistent and frequent bedwetting may be “22,221”. The model assumes that observed heterogeneity in responses is due to a latent (unobserved) grouping in the population.

The latent classes we derived describe typical and atypical development of nighttime bladder control: ‘normative development’ (71.5 % of the sample)—low probability of bedwetting at any time point; ‘infrequent delayed’ (14.3 %)—delayed attainment of nighttime bladder control (at around 6 years) and decreasing probability of infrequent bedwetting from 4 to 9 years; ‘infrequent persistent’ (8.6 %)—relatively high probability of infrequent bedwetting from 4 to 9 years; ‘frequent delayed’ (2.4 %)—high probability of frequent bedwetting at age 4 years, which decreased and became more infrequent at 6–9 years; ‘frequent persistent’ (3.2 %)—relatively high probability of bedwetting at least twice a week from 4 to 9 years.

### Difficult temperament

Mothers completed the Toddler Temperament Scale (TTS) [20] when the study children were 24 months old. The TTS comprises statements describing specific behaviours and mothers were asked to rate how often their child behaves in that way on a scale ranging from 1 (almost never) to 6 (almost always). The scale comprises nine temperament traits, but we restricted our analysis to five traits that were associated with bedwetting in an earlier study [21]. These included activity—higher activity level e.g. fidgeting during quiet activities; adaptability—difficulties adjusting to changes in routines; intensity—high-energy responses e.g. screaming, stamping feet; mood—unpleasant and fussy disposition and tendency to show anxiety when learning new tasks; persistence*—*more easily frustrated and likely to give up activities e.g. routine tasks such as dressing and picking up toys.

The Emotionality Activity Sociability (EAS) Questionnaire [22] was administered when study children were 38 months. The questionnaire comprises 20 statements about behaviours and mothers rated the extent to which each statement describes their child on a scale ranging from 1 (not at all like) to 5 (exactly like). Scores on these items are combined to form four subscales (each comprising five items): emotionality (tendency to show distress, e.g. cries easily, reacts intensely when upset), activity level (preferred level of activity, e.g. is always on the go, is very energetic), shyness (tendency to be inhibited with unfamiliar people, e.g. tends to be shy, takes a long time to warm up to strangers), and sociability (tendency to prefer the company of others, e.g. likes to be with people, prefers playing with others to playing alone). The factor structure of the EAS has been demonstrated in this sample to correspond well to these four traits and to have good stability over time [23].

### Psychological problems

Psychological problems were assessed when the child was aged 42 months using the Revised Rutter Parent Scale for Preschool Children, which is an extension of the Rutter behaviour scale [24]. The questionnaire comprises 43 statements describing behaviours and mothers are asked to rate the extent to which each statement describes their child on a scale comprising the options 1 (certainly true), 2 (sometimes true) and 3 (not true). Responses were aggregated to create scores in four domains: emotional difficulties (e.g. appears miserable, unhappy, tearful or distressed), conduct difficulties (e.g. fights with other children), hyperactivity (e.g. has poor concentration or short attention span) and prosocial behaviour (e.g. considerate of other people’s feelings). High levels of psychological problems are indicated by high scores on emotional difficulties, conduct difficulties and hyperactivity and low scores on the prosocial behaviour scale.

We show correlations between the Rutter, TTS and EAS subscales in Online Resource Table A.

### Confounders

We considered a range of potential confounders including gender and a number of socio-demographic measures derived from responses to a questionnaire, completed by mothers during the antenatal period, containing items on socioeconomic position and adversity. Binary variables were generated from these questions and each item was scored as 1 if an adversity was present and 0 if not. The items included social class based on the lower of the mother or partner’s occupational social class using the 1991 British Office of Population and Census Statistics classification and dichotomized into non-manual (professional, managerial or skilled professions) and manual (partly or unskilled occupations); early parenthood (<19 years versus ≥19 years), housing adequacy (yes/no—comprising crowding, periods of homelessness, living conditions, major defects/infestation), maternal education (defined as none versus high school qualifications or greater), major financial difficulties (yes/no), family size (<3 children versus ≥3 children) and the presence of a social network (yes/no—comprising emotional support, practical/financial support).

In addition we included developmental level, which was assessed at 18 months using a questionnaire developed by ALSPAC including items from the Denver Developmental Screening Test [25] and comprising four domains of development (fine motor, gross motor, communication and social skills). Scores on each domain were adjusted for age in weeks, standardised (using a linear regression model and extracting the residuals) and reversed where appropriate so that high values on all scores reflected a lower level of development. We adjusted for a total development score derived from the sum of the scores on each domain.

Finally, models were also adjusted for maternal depression using the Edinburgh Postnatal Depression Scale (EPDS) [26]. The EPDS was dichotomized at the standard cut-off (score >12) used to indicate probable depressive disorder [27]. The analysis with the TTS was adjusted for maternal depression at 21 months and the analyses with the EAS and Rutter Scale were adjusted for maternal depression at 33 months.

### Statistical modelling

We estimated the association between the risk factors and class membership using a series of univariable multinomial logistic regression models and employing the normative latent class as the baseline category for the outcome, before re-parameterizing to derive comparisons across the other outcome classes. Parameter estimates were obtained using the “Modal ML” 3-step method [28] implemented in Mplus. This has been shown to produce less-biased estimates than traditional three-step methods such as probability weighting, whilst avoiding the problem of covariates impacting on the measurement model itself [29]. Bias-adjusted estimates were obtained using the Mplus “auxiliary (r3step)” command. Models were adjusted for the confounders described above.

## Results

Bedwetting data were available for 10,810 children on at least one measurement occasion. Of these, 8761 had data from at least three time-points and 5849 had complete data. The proportion of children with bedwetting decreased over time and proportions did not change markedly when the sample was restricted to participants with more data—see [18]. We focused on the sample with bedwetting data available from at least three time-points (*n* = 8761) for the analyses presented here, but conclusions were consistent for the other two samples (available on request). Whilst the sample with complete bedwetting data had the lowest rates of socioeconomic disadvantage, there was little variation in the other confounders and the risk factors across samples (Table 1).

**Table 1:** Means and proportions of risk factors and confounders in the three samples considered for the analysis

	Complete data (*n* = 5849)	Partially missing data (*n* = 8761)	At least one bedwetting measure (*n* = 10,810)
Risk factors			
Toddler temperament traits			
Activity	23.1 (4.5)	23.13 (4.5)	23.2 (4.5)
Adaptability	12.7 (4.1)	12.77 (4.2)	12.8 (4.2)
Intensity	21.4 (4.6)	21.44 (4.6)	21.4 (4.6)
Mood	17.9 (5.6)	18.03 (5.7)	18.1 (5.7)
Persistence	16.3 (4.9)	16.31 (4.9)	16.3 (4.9)
EAS subscales			
Emotionality	14.2 (3.4)	14.23 (3.4)	14.2 (3.4)
Activity level	10.9 (2.5)	10.89 (2.5)	10.9 (2.5)
Sociability	10.3 (2.7)	10.27 (2.7)	10.3 (2.7)
Shyness	12.5 (4.1)	12.48 (4.1)	12.5 (4.1)
Rutter subscales			
Emotional difficulties	2.5 (1.7)	2.53 (1.7)	2.6 (1.7)
Conduct difficulties	3.5 (2.3)	3.59 (2.3)	3.6 (2.4)
Hyperactivity	2.6 (1.8)	2.62 (1.8)	2.6 (1.8)
Prosocial	6.7 (3.6)	6.67 (3.6)	6.7 (3.6)
Confounders			
Gender (male)	2959 (50.6 %)	4508 (51.5 %)	5580 (51.6 %)
Maturational level—total score	37.72 (5.5)	37.73 (5.6)	37.74 (5.7)
Socioeconomic variables			
Manual social class	705 (12.8 %)	1238 (15.4 %)	1661 (17.3 %)
Early parenthood <20 years	213 (3.64 %)	419 (4.8 %)	648 (6.0 %)
Housing inadequacy	408 (7.1 %)	748 (8.6 %)	982 (9.3 %)
Low maternal education	580 (10.0 %)	995 (11.6 %)	1342 (12.9 %)
Financial difficulties	803 (13.9 %)	1297 (15.1 %)	1653 (16.0 %)
Family size ≥3	251 (4.3 %)	424 (4.9 %)	600 (5.8 %)
Poor social network	716 (12.3 %)	1129 (13.0 %)	1433 (13.6 %)
Maternal depression			
21 months	5.11 (4.5)	5.27 (4.6)	5.34 (4.7)
33 months	5.37 (4.6)	5.57 (4.7)	5.65 (4.8)

Actual number with available data on each risk factor varies in each of the samples shown here

Manual social class includes manual and part/unskilled

### Association between the risk factors and bedwetting latent classes

We show the distribution of confounding variables in each latent class in Online Resource Table B. There were a higher proportion of males in each of the atypical latent classes. The frequent persistent class had the highest proportion of manual social class, housing inadequacy and low maternal education. The frequent delayed and frequent persistent classes had the highest proportion from larger families.

Table 2 presents the results of the analysis examining the associations between the risk factors and latent class membership. Odds ratios for these analyses were derived in relation to the normative latent class of nighttime bladder control, which was used as the reference group in this analysis. The results show the increase in odds of membership to each latent class per one standard deviation (SD) increase in the score for each risk factor.

**Table 2:** Odds ratios and 95 % confidence intervals for the association between the risk factors and latent class membership (*n* = 8761)

	Infrequent delayed	Infrequent persistent	Frequent delayed	Frequent persistent
(a) Temperament traits at 24 months (TTS)				
Activity				
Unadjusted				
8243	1.13 (1.03,1.24), ***p*** **=** **0.013**	1.10 (1.00, 1.21), ***p*** **=** **0.044**	1.27 (1.03, 1.57), ***p*** **=** **0.027**	1.15 (0.99, 1.32), *p* = 0.062
Adjusted				
6978	1.10 (0.99, 1.22), *p* = 0.068	1.05 (0.95, 1.17), *p* = 0.321	1.25 (1.01, 1.56), ***p*** **=** **0.041**	1.09 (0.93, 1.28) *p* = 0.282
Adaptability				
Unadjusted				
8211	1.24 (1.13, 1.36), ***p*** **<** **0.001**	1.19 (1.10, 1.30), ***p*** **<** **0.001**	1.25 (1.03, 1.51), ***p*** **=** **0.021**	1.33 (1.14, 1.55), ***p*** **<** **0.001**
Adjusted				
6957	1.18 (1.06, 1.31), ***p*** **=** **0.002**	1.17 (1.07, 1.30), ***p*** **<** **0.001**	1.14 (0.91, 1.41), *p* = 0.247	1.25 (1.05, 1.47), ***p*** **=** **0.010**
Intensity				
Unadjusted				
8238	1.17 (1.06, 1.29), ***p*** **=** **0.002**	1.16 (1.05, 1.28), ***p*** **=** **0.002**	1.09 (0.91, 1.30), *p* = 0.366	1.18 (1.02, 1.37), ***p*** **=** **0.002**
Adjusted				
6975	1.13 (1.01, 1.25), ***p*** **=** **0.029**	1.17 (1.05, 1.30), ***p*** **=** **0.004**	1.01 (0.83, 1.23), *p* = 0.915	1.21 (1.03, 1.42), ***p*** **=** **0.021**
Mood				
Unadjusted				
8238	1.16 (1.06, 1.28), ***p*** **<** **0.001**	1.15 (1.05, 1.26), ***p*** **=** **0.002**	1.13 (0.92, 1.39), *p* = 0.252	1.27 (1.10, 1.47), ***p*** **<** **0.001**
Adjusted				
6981	1.14 (1.03, 1.27), ***p*** **=** **0.012**	1.15 (1.04, 1.28), ***p*** **=** **0.007**	0.99 (0.76, 1.28), *p* = 0.936	1.23 (1.05, 1.46), ***p*** **=** **0.012**
Persistence				
Unadjusted				
8238	1.16 (1.05, 1.28), ***p*** **=** **0.003**	1.19 (1.08, 1.32), ***p*** **<** **0.001**	1.20 (0.99, 1.46), *p* = 0.062	1.25 (1.11, 1.41), ***p*** **<** **0.001**
Adjusted				
6977	1.09 (0.98, 1.22), *p* = 0.123	1.15 (1.03, 1.29), ***p*** **=** **0.015**	1.06 (0.87, 1.31), *p* = 0.561	1.10 (0.95, 1.27), *p* = 0.070
(b) Temperament traits at 38 months (EAS)				
Emotionality				
Unadjusted				
8123	1.02 (0.99, 1.05), *p* = 0.136	1.03 (1.00, 1.06), ***p*** **=** **0.036**	1.04 (0.98, 1.10), *p* = 0.167	1.00 (0.96, 1.04), *p* = 0.982
Adjusted				
6786	1.01 (0.98, 1.05), *p* = 0.414	1.04 (1.01, 1.08), ***p*** **=** **0.013**	1.01 (0.95, 1.07), *p* = 0.816	1.02 (0.97, 1.07), *p* = 0.510
Activity level				
Unadjusted				
8123	1.00 (0.97, 1.04), *p* = 0.851	1.02 (0.99, 1.05), *p* = 0.250	1.02 (0.96, 1.09), *p* = 0.438	1.03 (0.98, 1.09), *p* = 0.209
Adjusted				
6834	1.00 (0.96, 1.04), *p* = 0.983	1.03 (0.99, 1.07), *p* = 0.119	1.03 (0.97, 1.10), *p* = 0.357	1.04 (0.98, 1.10), *p* = 0.216
Sociability				
Unadjusted				
8123	1.03 (1.00, 1.07), *p* = 0.079	1.04 (1.01, 1.09), ***p*** **=** **0.015**	1.09 (1.03, 1.16), ***p*** **=** **0.005**	1.05 (1.00, 1.10), *p* = 0.064
Adjusted				
6679	1.02 (0.98, 1.06), *p* = 0.262	1.04 (1.00, 1.08), ***p*** **=** **0.047**	1.07 (1.00, 1.14), *p* = 0.059	1.03 (0.98, 1.09), *p* = 0.197
Shyness				
Unadjusted				
8158	1.00 (0.98, 1.02), *p* = 0.954	1.02 (0.99, 1.05), *p* = 0.132	1.00 (0.95, 1.04), *p* = 0.860	0.98 (0.94, 1.01), *p* = 0.195
Adjusted				
6814	0.99 (0.97, 1.02), *p* = 0.595	1.02 (0.99, 1.05), *p* = 0.113	1.00 (0.95, 1.05), *p* = 0.939	0.98 (0.94, 1.02), *p* = 0.247
(c) Psychological problems at 42 months (revised Rutter scale)				
Emotional difficulties				
Unadjusted				
8299	1.03 (0.94, 1.14), *p* = 0.529	1.17 (1.07, 1.28), ***p*** **<** **0.001**	1.12 (0.95, 1.33), *p* = 0.174	1.10 (0.95, 1.27), *p* = 0.205
Adjusted				
6878	1.01 (0.91, 1.13), *p* = 0.122	1.18 (1.07, 1.30), ***p*** **<** **0.001**	1.11 (0.92, 1.34), *p* = 0.270	1.07 (0.91, 1.26), *p* = 0.436
Conduct difficulties				
Unadjusted				
8299	1.32 (1.21, 1.45), ***p*** **<** **0.001**	1.30 (1.19, 1.41), ***p*** **<** **0.001**	1.19 (1.00, 1.42), *p* = 0.057	1.43 (1.25, 1.63), ***p*** **<** **0.001**
Adjusted				
6878	1.28 (1.15, 1.42), ***p*** **<** **0.001**	1.23 (1.12, 1.36), ***p*** **<** **0.001**	1.09 (0.89, 1.34), *p* = 0.390	1.26 (1.07, 1.48), ***p*** **=** **0.006**
Hyperactivity				
Unadjusted				
8299	1.21 (1.11, 1.33), ***p*** **<** **0.001**	1.18 (1.08, 1.30), ***p*** **<** **0.001**	1.29 (1.09, 1.54), ***p*** **=** **0.004**	1.29 (1.11, 1.50), ***p*** **<** **0.001**
Adjusted				
6878	1.13 (1.02, 1.26), ***p*** **=** **0.025**	1.15 (1.04, 1.28), ***p*** **=** **0.006**	1.17 (0.98, 1.41), *p* = 0.086	1.20 (1.01, 1.43), ***p*** **=** **0.033**
Low levels of prosocial behaviour				
Unadjusted				
8299	1.20 (1.09, 1.32), ***p*** **<** **0.001**	1.21 (1.10, 1.33), ***p*** **<** **0.001**	1.52 (1.26, 1.84), ***p*** **<** **0.001**	1.32 (0.83, 2.10), *p* = 0.185
Adjusted				
6878	1.13 (1.01, 1.26), ***p*** **=** **0.033**	1.07 (0.96, 1.19), *p* = 0.227	1.22 (1.00, 1.49), *p* = 0.053	1.18 (1.01, 1.38), ***p*** **=** **0.035**

Reference class = normative

Odds ratios for these analyses were derived in relation to the normative latent class of nighttime bladder control, which was used as the reference group. All models adjusted for gender, maturational level, socio-demographic factors and maternal depression

#### Early temperament

There was evidence that children rated by parents as having difficult temperament at 24 months on the TTS were more likely to experience atypical development of nighttime bladder control—Table 2a. All of the temperament traits were associated with increased odds of membership to the infrequent delayed, infrequent persistent and frequent persistent classes in the unadjusted model. The temperament trait of adaptability showed the strongest associations with these bedwetting classes. Odds ratios were generally highest for the frequent persistent class. For instance, a one SD increase in the adaptability score was associated with a 33 % (14–55 %) increase in the odds of belonging to the frequent persistent class compared with the normative class. Adjustment led to attenuation of these effects, but there was still evidence that adaptability, intensity  and mood at 24 months were associated with increased odds of bedwetting at 4–9 years. In the adjusted models, activity was only associated with membership to the frequent delayed class and persistence was associated with the infrequent persistent class. The most influential confounders were gender and developmental level. Activity was the only temperament trait associated with increased odds of membership to the frequent delayed class.

In contrast to the findings with the TTS, there was little evidence that scores on the EAS temperament subscales at 38 months were associated with increased odds of membership to the atypical latent classes—Table 2b. There was only weak evidence that the traits ‘emotionality’ and ‘sociability’ were associated with increased odds of membership to the infrequent persistent class.

#### Psychological problems

There was evidence that children rated by parents as having psychological problems on the revised Rutter scale at 42 months were more likely to experience bedwetting at 4–9 years—Table 2c. The strongest associations were found for conduct difficulties and odds ratios were highest for the frequent persistent class. A one SD increase in the conduct score led to increased odds of membership to the atypical classes ranging from a 30 % increase (infrequent persistent) to a 43 % increase (frequent persistent). There was strong evidence that hyperactivity was associated with increased odds of membership to all the atypical classes with odds ratios being highest in the frequent delayed and frequent persistent classes. A one SD increase in the hyperactivity score was associated with a 29 % increase in the odds of membership to these bedwetting classes. Low levels of prosocial behaviour were also linked to increased odds of membership to the infrequent delayed and frequent persistent classes in the adjusted model. With the exception of the infrequent persistent class, there was little evidence that increased levels of emotional difficulties at 42 months were associated with bedwetting at 4–9 years.

Adjustment led to attenuation of these effects, but there was still evidence that conduct problems, hyperactivity and low levels of prosocial behaviour were associated with atypical development of nighttime bladder control (but not for the frequent delayed class). The most influential confounders were gender and developmental level. Adjustment for the confounders had little effect on the odds ratio for the association between emotional difficulties and membership to the infrequent persistent class.

We additionally re-parameterized our regression models, comparing the persistent versus delayed classes and frequent versus infrequent classes, to examine whether there are specific risk factors that distinguish between different patterns of atypical wetting. With the exception of some evidence that the odds of emotional difficulties were higher in the infrequent persistent class compared with the infrequent delayed class, there were no differences between the atypical latent classes in their associations with the risk factors (results available on request).

## Discussion

### Main findings

We found evidence that difficult temperament and behaviour problems in early childhood are risk factors for bedwetting at school age. Difficult temperament traits at 24 months, including problems adapting to change, high intensity and negative mood, were associated with bedwetting at 4–9 years. Behaviour problems including conduct problems, hyperactivity and low levels of prosocial behaviour at 42 months were also associated with subsequent bedwetting. We found less evidence that early emotional difficulties are linked to bedwetting. The ‘infrequent persistent’ bedwetting class was the only pattern of bedwetting that was linked to emotional difficulties. This could be because this pattern of intermittent wetting is closest to what is often referred to as secondary enuresis or relapse. Children who are prone to emotional difficulties may be more susceptible to increased life stress and this could contribute to relapses in bedwetting [16].

We found little evidence that bedwetting is associated with temperament ratings of high activity level, but higher scores on the hyperactivity scale of the Rutter were associated with bedwetting. This apparently contradictory result could be explained by evidence that inattentive symptoms of ADHD are more common in children with bedwetting than hyperactive-impulsive symptoms [30, 31]. Two of the four items on the Rutter Hyperactivity scale refer to inattention (“Has poor concentration, or short attention span”; “Is inattentive”) whilst the items of the TTS and EAS activity scales describe the child’s activity level e.g. fidgeting and inability to sit still (TTS); energetic, always on the go (EAS). A previous study with the ALSPAC cohort found that the EAS activity scale at 38 months was associated with the hyperactive–impulsive type of ADHD at 7 years, but not the inattentive type [32].

### Potential mechanisms explaining the association between psychological factors and bedwetting

Our findings provide evidence that psychological factors in early childhood, including aspects of difficult temperament and behaviour problems, precede problems attaining nighttime bladder control. There are several potential explanations for this finding.

#### Common underlying cause of psychological problems and bedwetting: (a) neurobiological deficit

The association between difficult temperament/psychological problems and bedwetting could be due to a common underlying neurobiological deficit. Temperament has been defined as constitutional differences in reactivity (biological arousability) and self-regulation, and is believed to have a neurobiological basis [33]. Support for a common underlying neurobiological basis for bedwetting and psychological problems comes from the widely reported link between ADHD and bedwetting [34]. The high co-occurrence of enuresis and ADHD is based on complex neural networks including cortical, subcortical and brainstem regions. A deficit in inhibitory control is believed to be central to ADHD and is also involved in the pathophysiology of nocturnal enuresis—a deficit in the basic inhibitory function of the brainstem leads to lack of arousal and the inability to inhibit the micturition reflex during sleep [11].

#### (b) Family adversity and stress

There is evidence that exposure to family adversity and stress in early childhood could contribute to an increased risk of psychological problems [35] and could also interfere with learning bladder control [16, 36]. The stress hormone cortisol exerts a negative feedback directly on the pituitary and also on the synthesis and secretion of antidiuretic hormone, a lack of which is associated with bedwetting due to the production of more urine than the bladder can hold [37, 38]. It is notable; however, that even after adjusting for a range of factors associated with family adversity we still found evidence for associations between difficult temperament/psychological problems and bedwetting.

#### (c) Parenting style

It is also possible that parental attitudes and discipline are a common underlying cause of temperament/psychological difficulties and bedwetting. Parenting has been linked to the development of difficult temperament and behaviour problems [39]. Certain parenting styles, such as inconsistent scheduling of activities and lack of responsiveness to the child’s physical needs, could lead to ineffective toilet training strategies. It is, however, difficult to disentangle shared genetic influences leading to similar temperament traits in parents and offspring, from environmental effects of parenting style on child development.

#### Difficulties in toilet training

The age of 2–3 years is a sensitive phase for acquiring bladder control and this is when the majority of parents initiate toilet training [16]. This is a stressful transition period for both parents and children and is likely to be especially challenging for parents of children with difficult temperament and psychological problems. An early study found that low adaptability, high intensity and negative mood clustered together to form a ‘difficult child’ construct [40]. Attempts at toilet training a child who finds it difficult to adapt to changes in their toileting routine may provoke intense negative reactions and anxiety, which could interfere with the acquisition of bladder control and make children vulnerable to problems attaining continence. Negative parental reactions could lead to anxiety and stress, which could interfere with the acquisition of bladder control [41].

The pathways through which difficult temperament and psychological problems in early childhood are linked to bedwetting are likely to be highly complex and to involve a combination of the mechanisms described above. In showing longitudinal associations between early psychological problems and subsequent bedwetting, we are not providing evidence against an association in the other direction. For instance, emotional symptoms could emerge when children become aware that bedwetting is unusual for their age, or in the face of negative reactions from their parents or peers. Psychological factors are also likely to be influential in maintaining bedwetting and contributing to relapses.

### Strengths and limitations

A major strength of our study is the prospective design with data available on difficult temperament and psychological problems before the age at which most children attain nighttime bladder control. This addresses potential biases inherent in cross-sectional studies and provides evidence that these early risk factors are associated with subsequent problems attaining nighttime bladder control.

A possible limitation of this study is the use of maternal reports for psychological factors. There is evidence to suggest that maternal depression results in a tendency view their child negatively and over-report problem behaviours [42]. Relying on maternal reports could have resulted in misclassification of difficult temperament or psychological problems in their young children. However, any such misclassification is likely to have been non-differential with respect to our outcome.

It has been argued that direct observations in a laboratory setting are more objective measures of child temperament than maternal reports [43]. A single observation from an artificial setting, however, could also have limitations by providing only a snapshot of the child’s current state. Parental reports of temperament traits are more likely to reflect more stable behavioural characteristics over multiple settings [33].

There was a relative lack of evidence for associations between the risk factors and the frequent delayed class. This might be due to this being the smallest class, leading to larger standard errors and hence, a lack of precision in our estimates of the effect of the risk factors on membership to this class.

### Implications and conclusions

There is evidence that children who suffer from frequent bedwetting are more likely to experience persistent incontinence than those with less frequent bedwetting. A previous study found a greater proportion of frequent, compared with infrequent, bedwetters among older children and adolescents [44]. Frequent bedwetting has been linked to persistent continence problems into adult life [45]. It would be particularly useful to be able to identify factors that might distinguish between children who are at risk of persistent bedwetting from those who are likely to experience a natural resolution of their bedwetting. This knowledge could improve the identification of children who should be prioritised for specialist services. We did not find evidence for distinct aetiologies for the latent classes. Our findings were mostly in agreement with a dose response relationship, in which increasing levels of difficult temperament and behaviour problems in early childhood are associated with increasing severity (frequency and persistence) of bedwetting. This could indicate that these risk factors are common to all types of atypical development of nighttime bladder control.

There is evidence that severe (frequent and persistent) bedwetting is often accompanied by daytime wetting [44] and, in agreement with this, we previously found the highest percentage of daytime wetting in the frequent persistent class [18]. It is possible that distinct risk factors would emerge if we further refined our bedwetting latent classes by incorporating additional symptom information such as concurrent daytime wetting and indicators of bladder dysfunction. For instance, psychological factors may be differentially associated with combined (day and night) wetting compared with bedwetting alone.

Many parents do not consider seeking treatment for bedwetting until it has started to have secondary impacts on their child’s quality of life and many are unaware that there are effective treatments available [46]. In the UK, evaluation and treatment for bedwetting is generally considered for children aged 5 and over. Parents should be encouraged to seek treatment for children who are still wetting the bed frequently at 5 years because timely intervention could help to reduce the risk of continence problems becoming persistent and lower the risk of secondary impacts.

## Electronic supplementary material

Below is the link to the electronic supplementary material.

Supplementary material 1 (DOCX 59 kb)

## References

[1] Byrd RS, Weitzman M, Lanphear NE, Auinger P (1996). Bed-wetting in US children: epidemiology and related behaviour problems. Pediatrics.

[2] Fergusson DM, Horwood LJ, Shannon FT (1986). Factors related to the age of attainment of nighttime bladder control: an 8-year longitudinal study. Pediatrics.

[3] Butler RJ, Golding J, Northstone K (2005). Nocturnal enuresis at 7.5 years old: prevalence and analysis of clinical signs. Br J Urol Int.

[4] Austin PF, Bauer S, Bower W, Chase J, Franco I, Hoebeke P, Rittig S, Vande Walle J, von Gontard A, Wright A, Yang A, Nevéus T (2014). The standardization of terminology of bladder function in children and adolescents: update report from the Standardization Committee of the International Children’s Continence Society (ICCS). J Urol.

[5] Joinson C, Heron J, Emond A, Butler R (2007). Psychological problems in children with bedwetting and combined (day and night) wetting: a UK population-based study. J Pediatr Psychol.

[6] Butler RJ, McKenna S (2002). Overcoming parental intolerance in childhood nocturnal enuresis: a survey of professional opinion. BJU Int.

[7] Shaffer D (1973) The association between enuresis and emotional disorder: a review of the literature. In: Kolvin I, Mac Keith RC, Meadow SR (eds) Bladder control and enuresis. William Heinemann Medical Books, London, pp 137–147

[8] Barbour AF, Boyd MM, Borland EM, Miller S, Oppel TE (1963). Enuresis as a disorder of development. Br Med J.

[9] Jarvelin MR (1989). Developmental history and neurobiological findings in enuretic children. Dev Med Child Neurol.

[10] Baker BL, McIntyre LL, Blacher J, Crnic K, Edelbrock C, Low C (2003). Pre-school children with and without developmental delay: behaviour problems and parenting stress over time. J Intellect Disabil Res.

[11] von Gontard A, Equit M (2015). Comorbidity of ADHD and incontinence in children. Eur Child Adolesc Psychiatry.

[12] Butler RJ (1998). Annotation: night wetting in children: psychological aspects. J Child Psychol Psychiatry.

[13] Longstaffe S, Moffatt ME, Whalen JC (2000). Behavioural and self-concept changes after six months of enuresis treatment: a randomized, controlled trial. Pediatrics.

[14] Moffatt ME (1989). Nocturnal enuresis: psychologic implications of treatment and nontreatment. J Pediatr.

[15] McGee R, Makinson T, Williams S, Simpson A, Silva PA (1984). A longitudinal study of enuresis from five to nine years. Aust Paediatr J.

[16] Jarvelin MR, Moilanen I, Vikevainen-Tervonen L, Huttunen NP (1990). Life changes and protective capacities in enuretic and non-enuretic children. J Child Psychol Psychiatry.

[17] Muris P, Ollendick TH (2005). The role of temperament in the etiology of child psychopathology. Clin Child Fam Psychol Rev.

[18] Sullivan S, Joinson C, Heron J (2015) Factors predicting atypical development of nighttime bladder control: a prospective cohort study (under review) 10.1097/DBP.0000000000000229 PMC4631123 26468941

[19] Boyd A, Golding J, Macleod JAA, Lawlor DA, Fraser A, Henderson AJW, Molloy L, Ness A, Ring S, Smith GD (2013). Cohort profile: the ‘children of the 90s’–the index offspring of the Avon Longitudinal Study of Parents and Children. Int J Epidemiol.

[20] Fullard W, McDevitt SC, Carey WB (1984). Assessing temperament in one-to-threeyear-old children. J Pediatr Psychol.

[21] Joinson C, Heron J, Butler R, Croudace T (2009). Development of nighttime bladder control from 4–9 years: association with dimensions of parent rated child maturational level, child temperament and maternal psychopathology. Longitud Life Course Stud.

[22] Buss A, Plomin R (1984). Temperament: early developing personality traits.

[23] Bould H, Joinson C, Sterne J, Araya R (2013). The emotionality activity sociability temperament survey: factor analysis and temporal stability in a longitudinal cohort. Personal Individ Differ.

[24] Elander J, Rutter M (1996). Use and development of the Rutter parents’ and teachers’ scales. Int J Methods Psychiatr Res.

[25] Frankenburg WK, Dodds J, Archer P, Shapiro H, Bresnick B (1992). The Denver II: a major revision and restandardization of the Denver Developmental Screening Test. Pediatrics.

[26] Cox JL, Holden JM, Sagovsky R (1987). Detection of postnatal depression. Development of the 10-item Edinburgh Postnatal Depression Scale. Br J Psychiatry.

[27] Evans J, Heron J, Francomb H, Oke S, Golding J, ALSPAC Study Team (2001). Cohort study of depressed mood during pregnancy and after childbirth. Br Med J.

[28] Vermunt JK (2010). Latent class modeling with covariates: two improved three-step approaches. Political Anal.

[29] Asparouhov T, Muthen BO (2013) Auxiliary variables in mixture modeling: 3-step approaches using Mplus. Mplus Web Notes: No. 15. http://www.statmodel.com/examples/webnotes/webnote15.pdf. Accessed 19th May 2015

[30] Baeyens D, Roeyers H, Hoebeke P, Verte S, Van Hoecke E, Walle JV (2004). Attention deficit/hyperactivity disorder in children with nocturnal enuresis. J Urol.

[31] Elia J, Takeda T, Deberardinis R, Burke J, Accardo J, Ambrosini PJ, Blum NJ, Brown LW, Lantieri F, Berrettini W, Devoto M, Hakonarson H (2009). Nocturnal enuresis: a suggestive endophenotype marker for a subgroup of inattentive attention-deficit/hyperactivity disorder. J Pediatr.

[32] Stringaris A, Maughan B, Goodman R (2010). What’s in a disruptive disorder? Temperamental antecedents of oppositional defiant disorder: findings from the Avon Longitudinal Study. J Am Acad Child Adolesc Psychiatry.

[33] Rothbart MK, Bates JE (2006) Temperament. In: Damon W, Lerner R, Eisenberg N (eds) Handbook of child psychology. Social, emotional, and personality development, vol 3, 6th edn. Wiley, New York, pp 99–166

[34] Baeyens D, Roeyers H, Van Erdeghem S, Hoebeke P, Vande Walle J (2007). The prevalence of attention deficit-hyperactivity disorder in children with nonmonosymptomatic nocturnal enuresis: a 4-year followup study. J Urol.

[35] Shaw DS, Vondra JI, Hommerding KD, Keenan K, Dunn M (1994). Chronic family adversity and early child behaviour problems: a longitudinal study of low income families. J Child Psychol Psychiatry.

[36] Rutter M, Yule,W, Graham P (1973) Enuresis and behavioural deviance: some epidemiological considerations. In: Kolvin I, Mac Keith RC, Meadow SR (eds) Bladder control and enuresis. William Heinemann Medical Books, London, pp 137–147

[37] Aikawa T, Kasahara T, Uchiyama M (1998). The arginine–vasopressin section profile of children with primary nighttime enuresis. Eur Urol.

[38] Rittig S, Knudsen UB, Norgaard JP, Pedersen EB, Djurhuus JC (1989). Abnormal diurnal rhythm of plasma vasopressin and urinary output in patients with enuresis. Am J Physiol.

[39] Lavigne JV, Gibbons RD, Christoffel KK, Arend R, Rosenbaum D, Binns H, Dawson N, Sobel H, Isaacs C (1996). Prevalence rates and correlates of psychiatric disorders among preschool children. J Am Acad Child Adolesc Psychiatry.

[40] Thomas A, Chess S, Birch HG, Hertzig ME, Korn S (1963). Behavioural individuality in early childhood.

[41] Houts AC (1991). Nighttime enuresis as a bio-behavioural problem. Behav Ther.

[42] Fergusson DM, Lynskey MT, Horwood LJ (1993). The effect of maternal depression on maternal ratings of child behaviour. J Abnorm Child Psychol.

[43] Durbin CE, Hayden EP, Klein DN, Olino T (2007). Stability of laboratory assessed temperament traits from ages 3 to 7. Emotion.

[44] Yeung CK, Sreedhar B, Sihoe JD, Sit FK, Lau J (2006). Differences in characteristics of nighttime enuresis between children and adolescents: a critical appraisal from a large epidemiological study. Br J Urol Int.

[45] Yeung CK, Sihoe JD, Sit FK, Bower W, Sreedhar B, Lau J (2004). Characteristics of primary nocturnal enuresis in adults: an epidemiological study. BJU Int.

[46] Schlomer B, Rodriguez E, Weiss D, Copp H (2013) Parental beliefs about nocturnal enuresis causes, treatments, and the need to seek professional medical care. J Pediatr Urol 9(6 Pt B):1043–1048 10.1016/j.jpurol.2013.02.013 PMC4648250 23608323

