# Supplementary material for: Early childhood psychological factors and risk for bedwetting at school age in a UK cohort
Source: Eur Child Adolesc Psychiatry. 2015 Aug 21;25:519–28. doi: 10.1007/s00787-015-0756-7 (PMC4854940; doi:10.1007/s00787-015-0756-7)
Supplement: Supplementary file 1 — Supplementary material 1 (DOCX 59 kb) [file 787_2015_756_MOESM1_ESM.docx]

Electronic supplementary material for the article entitled:

Early childhood psychological factors and risk for bedwetting at school age in a UK cohort

Carol Joinson PhD^1^, Sarah Sullivan PhD^1,2^, Alexander von Gontard MD PhD^3^, Jon Heron PhD^1^

**Affiliations**:

1. School of Social and Community Medicine, University of Bristol, Oakfield House, Oakfield Grove, Clifton, Bristol BS8 2BN, UK.
2. Epidemiology and Health Services Research, CLAHRC West, Lewins Mead, Bristol.
3. Department of Child and Adolescent Psychiatry, Saarland University Hospital, 66421 Homburg, Germany.

**Email address of corresponding author:** [Carol.Joinson@bristol.ac.uk](mailto:Carol.Joinson@bristol.ac.uk)

Supplementary table A: Correlations between the Rutter, TTS, EAS subscales

|  | **Rutter** | | | | **TTS** | | | | | **EAS** | | | |
| --- | --- | --- | --- | --- | --- | --- | --- | --- | --- | --- | --- | --- | --- |
|  | Emotional | Conduct | Hyperactivity | Prosocial | Activity | Adaptability | Intensity | Mood | Persistence | Emotionality | Activity level | Shyness | Sociability |
| **Rutter** |  |  |  |  |  |  |  |  |  |  |  |  |  |
| Emotional | 1.000 |  |  |  |  |  |  |  |  |  |  |  |  |
| Conduct | 0.226 | 1.000 |  |  |  |  |  |  |  |  |  |  |  |
| Hyperactivity | 0.219 | 0.382 | 1.000 |  |  |  |  |  |  |  |  |  |  |
| Prosocial | 0.150 | 0.270 | 0.171 | 1.000 |  |  |  |  |  |  |  |  |  |
| **TTS** |  |  |  |  |  |  |  |  |  |  |  |  |  |
| Actvity | 0.024 | 0.243 | 0.342 | 0.058 | 1.000 |  |  |  |  |  |  |  |  |
| Adaptability | 0.152 | 0.291 | 0.260 | 0.242 | 0.477 | 1.000 |  |  |  |  |  |  |  |
| Intensity | 0.124 | 0.244 | 0.224 | 0.056 | 0.419 | 0.399 | 1.000 |  |  |  |  |  |  |
| Mood | 0.287 | 0.215 | 0.191 | 0.246 | 0.264 | 0.585 | 0.378 | 1.000 |  |  |  |  |  |
| Persistence | 0.084 | 0.200 | 0.282 | 0.237 | 0.277 | 0.340 | 0.136 | 0.281 | 1.000 |  |  |  |  |
| **EAS** |  |  |  |  |  |  |  |  |  |  |  |  |  |
| Emotionality | 0.370 | 0.206 | 0.189 | 0.098 | 0.116 | 0.212 | 0.261 | 0.314 | 0.137 | 1.000 |  |  |  |
| Activity level | 0.110 | -0.075 | -0.220 | 0.096 | -0.275 | -0.053 | -0.125 | 0.077 | 0.017 | -0.004 | 1.000 |  |  |
| Shyness | 0.334 | -0.044 | -0.072 | 0.194 | -0.127 | 0.103 | -0.040 | 0.258 | 0.032 | 0.101 | 0.297 | 1.000 |  |
| Sociability | 0.291 | 0.017 | -0.022 | 0.216 | -0.131 | 0.089 | -0.042 | 0.191 | 0.039 | -0.012 | 0.297 | 0.567 | 1.000 |

Supplementary Table B: Mean (SD) of scores on risk factors and distribution of confounding variables in the bedwetting latent classes

|  | Normative | Infrequent delayed | Infrequent persistent | Frequent delayed | Frequent persistent | p-value |
| --- | --- | --- | --- | --- | --- | --- |
| *Gender* |  |  |  |  |  |  |
| Male | 3018 (46.8%) | 641 (60.1%) | 509 (66.6%) | 137 (65.9%) | 203 (72.5%) | p<0.001 |
| Female | 3425 (53.2%) | 425 (39.9%) | 255 (33.4%) | 71 (34.1%) | 77 (27.5%) |  |
|  |  |  |  |  |  |  |
| *Social class* |  |  |  |  |  |  |
| Manual | 912 (15.5%) | 135 (13.8%) | 105 (14.9%) | 24 (12.6%) | 62 (23.8%) | p=0.002 |
| Non-manual | 4977 (84.5%) | 843 (86.2%) | 599 (85.1%) | 166 (87.4%) | 198 (76.2%) |  |
|  |  |  |  |  |  |  |
| *Early parenthood* |  |  |  |  |  |  |
| Yes | 307 (4.8%) | 48 (4.5%) | 39 (5.1%) | 9 (4.3%) | 16 (5.7%) | p=0.911 |
| No | 6136 (95.2%) | 1018 (95.5%) | 725 (94.9%) | 199 (95.7%) | 264 (94.3%) |  |
|  |  |  |  |  |  |  |
| *Housing inadequacy* | |  |  |  |  |  |
| Yes | 531 (8.3%) | 89 (8.4%) | 66 (8.7%) | 22 (10.7%) | 40 (14.4%) | p=0.008 |
| No | 5869 (91.7%) | 967 (91.6%) | 690 (91.3%) | 184 (89.3%) | 238 (85.6%) |  |
|  |  |  |  |  |  |  |
| *Low maternal education* |  |  |  |  |  |  |
| Yes | 754 (12.0%) | 99 (9.5%) | 72 (9.6%) | 23 (11.2%) | 47 (17.1%) | p=0.002 |
| No | 5529 (88.0%) | 947 (90.5%) | 677 (90.4%) | 183 (88.8%) | 228 (82.9%) |  |
|  |  |  |  |  |  |  |
| *Financial difficulties* | |  |  |  |  |  |
| Yes | 957 (15.1%) | 172 (16.5%) | 102 (13.7%) | 26 (12.6%) | 40 (14.6%) | p=0.443 |
| No | 5372 (84.9%) | 872 (83.5%) | 643 (86.3%) | 180 (87.4%) | 234 (85.4%) |  |
|  |  |  |  |  |  |  |
| *Family size* |  |  |  |  |  |  |
| >=3 | 295 (4.6%) | 50 (4.7%) | 42 (5.6%) | 16 (7.9%) | 21 (7.6%) | p=0.040 |
| <3 | 6054 (95.4%) | 1005 (95.3%) | 706 (94.4%) | 187 (92.1%) | 254 (92.4%) |  |
|  |  |  |  |  |  |  |
| *Poor social network* | |  |  |  |  |  |
| Yes | 797 (12.5%) | 143 (13.6%) | 111 (14.7%) | 35 (17.0%) | 43 (15.5%) | p=0.083 |
| No | 5600 (87.5%) | 911 (86.4%) | 645 (85.3%) | 171 (83.0%) | 235 (84.5%) |  |
